# Label-free Raman spectroscopy provides early determination and precise localization of breast cancer-colonized bone alterations†
†Electronic supplementary information (ESI) available. See DOI: 10.1039/c7sc02905e


**DOI:** 10.1039/c7sc02905e

**Published:** 2017-11-15

**Authors:** Chi Zhang, Paul T. Winnard Jr, Sidarth Dasari, Scott L. Kominsky, Michele Doucet, Swaathi Jayaraman, Venu Raman, Ishan Barman

**Affiliations:** a Department of Mechanical Engineering , Johns Hopkins University , Whiting School of Engineering , Latrobe Hall 103 , Baltimore , MD 21218 , USA . Email: ibarman@jhu.edu ; Tel: +1-410-516-0656; b Division of Cancer Imaging Research , Russell H. Morgan Department of Radiology and Radiological Science , Johns Hopkins University School of Medicine , 720 Rutland Avenue, Rm 340 Traylor Building , Baltimore , MD , USA 21205 . Email: vraman2@jhmi.edu ; Tel: +1-410-955-7492; c Indiana University School of Medicine , Indianapolis , IN , USA; d Department of Orthopaedic Surgery , Johns Hopkins University School of Medicine , Baltimore , MD , USA; e Department of Oncology , Johns Hopkins University School of Medicine , Baltimore , MD , USA

## Abstract

Breast neoplasms frequently colonize bone and induce development of osteolytic bone lesions by disrupting the homeostasis of the bone microenvironment. This degenerative process can lead to bone pain and pathological bone fracture, a major cause of cancer morbidity and diminished quality of life, which is exacerbated by our limited ability to monitor early metastatic disease in bone and assess fracture risk. Spurred by its label-free, real-time nature and its exquisite molecular specificity, we employed spontaneous Raman spectroscopy to assess and quantify early metastasis driven biochemical alterations to bone composition. As early as two weeks after intracardiac inoculations of MDA-MB-435 breast cancer cells in NOD-SCID mice, Raman spectroscopic measurements in the femur and spine revealed consistent changes in carbonate substitution, overall mineralization as well as crystallinity increase in tumor-bearing bones when compared with their normal counterparts. Our observations reveal the possibility of early stage detection of biochemical changes in the tumor-bearing bones – significantly before morphological variations are captured through radiographic diagnosis. This study paves the way for a better molecular understanding of altered bone remodeling in such metastatic niches, and for further clinical studies with the goal of establishing a non-invasive tool for early metastasis detection and prediction of pathological fracture risk in breast cancer.

## Abstract 2

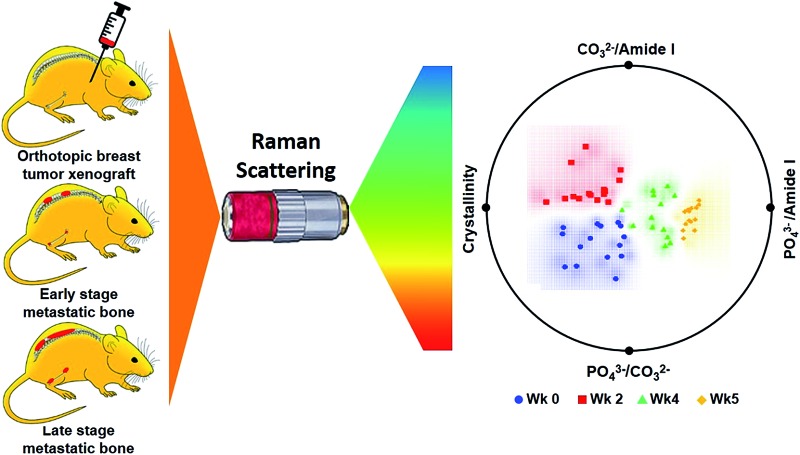
Raman spectral markers offer new routes to recognition of biomolecular alterations at sites of nascent and progressing metastatic cancer in bone.

## Introduction

In the United States, breast cancer remains the most common malignant neoplasm of women and it is estimated that ∼250 000 cases of female breast carcinoma *in situ* will be diagnosed along with an estimated 40 000 deaths in 2017.^1^ While local breast tumors respond very well to therapy, distant metastases are a frequent complication with a very poor response to therapy.^2^ The highest incidence of metastatic progression is to bone with the latter representing the first metastatic site in 30–40% of breast cancer patients,^3^,^4^ and bone metastases being reported in up to nearly 70% of patients during disease progression.^4^,^5^ There are three types of bone metastases: osteolytic, marked by heightened resorption that results in loss of bone mineral and matrix; osteoblastic that causes an increase in abnormal weakened bone formation; and mixed osteolytic/osteoblastic lesions.^6^ While osteolytic disease predominates,^7^ all three forms occur in the context of breast cancer.^8^ Bone colonization and dysregulation of the normal bone remodeling processes results in a range of skeletal related events (SREs), such as severe bone pain, hypercalcemia, ablation of bone marrow resulting in pancytopenia, spinal cord compression as well as pathological fractures.^5^,^6^,^8^,^9^ Such SREs often occur in load-bearing bones, for instance the neck of the femur or in the pelvis, which can present particular treatment challenges.

Given these circumstances, early accurate identification of patients at risk for bone metastasis is a critical need for the correct staging of patients and selection of appropriate therapeutic regimens.^6^,^10^ Current imaging technologies used to screen for bone metastatic lesions include ^99m^Tc based bone scintigraphy (BS), single photon emission computed tomography (SPECT), alone or combined with computed tomography (CT), CT combined with positron emission tomography (PET), and whole body magnetic resonance imaging (wbMRI).^10^ Advances in PET/CT have provided spatial resolutions in the 5 to 8 mm (full-width at half-maximum (FWHM)) range,^11^ which makes detection of small early lesions more likely. Nevertheless, such detection does not provide information about the bone's microstructural composition and whether it has been compromised.

To provide comprehensive management of bone metastases requires not only a determination of metastatic lesions but also an accurate assessment of fracture risk.^6^ Fracture risk needs to be assessed at the time of discovery as well as monitored during follow up of response to treatment, as a decrease in tumor burden alone does not ensure that a corresponding increase in bone integrity and strength has occurred.^12^ Risk prediction based on bone mineral density (BMD) using dual energy X-ray absorptiometry (DXA) has been a standard of practice. However, this methodology does not provide an accurate estimate of bone mineral content.^13^ Computed tomography (CT) is superior to 2D X-ray technologies for evaluating BMD and a recently developed CT-based structural rigidity analysis technology has demonstrated improved fracture risk prediction with two clinical trials showing 100% sensitivity but only fair, 60–70%, specificity.^12^,^14^ Numerous recent reports have discussed why an evaluation of BMD alone falls short of accurately gauging bone fragility by pointing out that the organic component of bone, in conjunction with bio-hydroxyapatite, contributes greatly to bone toughness.^15^–^18^ An accurate fracture risk assessment demands knowledge of the underlying biochemical matrix integrity along with the crystalline mineral composition of the bone. Hence, development of non-invasive technologies that can detect changes in bone matrix and mineral composition at early stages of colonization would meet a significant clinical need.

Label-free vibrational spectroscopy's unique attributes can address these unmet needs as it provides objective biomarkers of bone composition for diagnoses^16^,^18^ and may provide patient stratifications for more effective therapy.^17^ The exquisite molecular specificity of this approach enables multiplexed biomolecular analyses without necessitating exposure to radiation or addition of exogenous contrast agents.^19^,^20^ Furthermore, in comparison to IR spectroscopy, the higher spatial resolution and the ability to analyze fresh tissue specimen opens up tremendous opportunities for Raman scattering measurements. A recent review indicates the wide-ranging potential of Raman spectroscopy to assess the biochemical attributes of bone, which when combined with mechanical loading regimes, correlate with bone failure responses at the ultrastructural level.^21^ In addition, recent studies have expanded on this and demonstrated Raman spectroscopy's ability to detect specific pathological changes in bone matrix components, including alterations in phosphate, carbonate, the amide backbone of collagen, as well as collagen cross-link maturity, and use them in predictive models for fracture risk.^22^–^24^ Important insights can also be gained from the emerging evidence on Raman spectral changes in metastatic bone primed by prostate and breast cancer.^16^,^25^,^26^

**Fig. 1:**
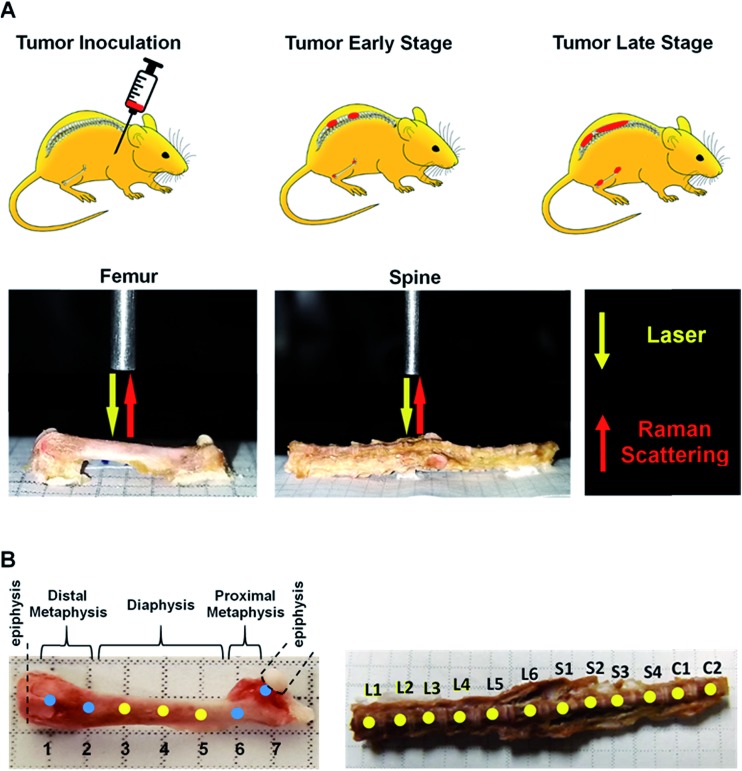
Schematic representation of experimental model of breast cancer bone metastasis and depiction of subsequent Raman spectroscopy measurements. (A) Intracardiac injected of breast cancer 435-tdT cells (top: left-hand panel) and ensuing metastases in the femur and spine as demarcated in red (top: central and right-hand panels). Raman microspectroscopy (bottom panels) was used to record spectra from these affected femurs and spines. (B) Raman spectra were collected at 2 mm intervals along the length of the femurs as indicated by numbered spots. Raman spectra of the spines were collected from central regions of lumbar (L1–L6), sacral (S1–S4), and caudal (C1–C2) vertebrae.

Here, we present results from a pilot study designed to determine whether Raman spectroscopy can detect changes in mineral and biomolecular components of bone early in metastatic progression. The above-cited studies on the use of Raman spectroscopy to evaluate bone matrix changes in metastatic model systems performed the assessments at mid-to-late stage disease, and a time-related assessment of bone quality alteration, particularly at early colonization, remains unexplored. We used a well-established bone metastatic model system where intracardiac injections of MDA-MB-435 breast cancer cells results in bone metastases.^27^–^29^ During this study, bone metastasis progression was tracked weekly for five weeks. Raman spectral evaluations were performed on *ex vivo* specimens of femurs and spines (Fig. 1). Relative to normal bone, Raman spectroscopy was able to detect changes in the biochemical characteristics of bones with metastatic lesions as early as two weeks after tumor cell inoculations while X-ray images showed no sign of disease even after 5 weeks of metastatic progression. Our findings demonstrate the feasibility of using Raman spectroscopy for early detection and localization of metastatic disease in bone by quantifying surrogate markers, notably changes in bone matrix composition, at the site of the disease. These early alterations in the intrinsic biochemical characteristics of bone suggest compromised integrity and a weakening of the bone. These findings pave the way for further investigations into pathological fracture risk estimation through noninvasive, spatially offset Raman spectroscopy measurements^30^ of bone in real time.

## Experimental

### Cell culture

The human breast cancer cell line, MDA-MB-435, was obtained from ATCC. The MDA-MB-435 cell line was established in 1976 from a pleural effusion from an untreated 31 year-old female diagnosed with adenocarcinoma of the breast.^31^ MDA-MB-435 cells were authenticated at the Johns Hopkins Genetic Resource Core Facility with the short tandem repeat marker results cross checked against cell lines at the ATCC bank. Generation and characterization of MDA-MB-435 engineered to constitutively express a bright red fluorescent protein: tdTomato (designated in the further discussion as 435-tdT cells), to facilitate *in vivo* and *ex vivo* tracking of metastatic progression, has been previously described.^32^ Cells were cultured in RPMI-10% FBS medium in a standard humidified incubator at 37 °C and 5% CO_2_.

### Intracardiac experimental bone metastasis model

All animal handling procedures were performed in accordance with protocols approved by the Johns Hopkins University Institutional Animal Care and Use Committee and conformed to the Guide for the Care and Use of Laboratory Animals published by the NIH. Non-diabetic severe combined immunodeficient (NOD-SCID) female mice were used. Four six-to-eight week old mice were anesthetized by intraperitoneal injections of a mixture of xylazine (11 mg kg^–1^) and ketamine (72 mg kg^–1^). Mice were successfully injected with 435-tdT cells (1 × 10^5^) in 0.1 ml of sterile RPMI-10% FBS through the intercostal space into the left ventricle of the heart using 26-gauge needle on a tuberculin syringe. Mice were sacrificed at different time points: on the day of tumor cell injection and at two, four and five weeks post tumor cell injection, by administering an overdose of anesthetic [saline : ketamine : acepromazine (2 : 1 : 1)] followed by cervical dislocation. Intact skeletons were then immediately dissected away from soft tissue, wrapped in phosphate buffered saline soaked gauze and then in aluminium foil and stored at –20 °C.

### Live animal optical and X-ray imaging and *ex vivo* fluorescence microscopy

Live animal optical imaging was done in a Xenogen IVIS® Spectrum (PerkinElmer) optical scanner under 2% isoflurane/O_2_ gas anesthesia, as previously described.^32^ The spectral unmixing tool in the Living Image® 4.2 software package was used to remove background autofluorescence. The unmixing tool also provided a means to focus on fluorescence from bone by masking the fluorescence from brain metastases that were also generated with this mouse model. X-ray images were captured using a Faxitron® MX-20 X-ray scanner (Faxitron X-ray Corp., Lincolnshire, IL).

Fluorescence microscopy was on an inverted Nikon ECLIPSE TS 100 microscope (Nikon Instruments, Inc., Melliville, NY) equipped with a Texas Red filter cube. The fluorescence light source was an X-Cite 120 Fluorescence Illumination System (Photonic Solutions, Inc., Edinburgh, UK). A 2× objective and 2s exposure time were used. Anterior and posterior photomicrographs of each end of the femurs were obtained using a Photometrics CoolSnap™ ES (Roper Scientific, Trenton, NJ) camera interfaced with NIS-Elements F3.2 software. Fluorescence intensities of approximately equal sized ROIs of the anterior and posterior metaphysis regions, which excluded the non-fluorescing diaphysis, were quantitated with ImageJ. Images of proximal and distal metaphysis, which contained overlapping portions of each diaphysis, were stitched together during reconstruction.

### Raman spectroscopy

Intact femurs and spines harvested from control mice without tumors and 435-tdT tumor-bearing mice were placed on a flat aluminum substrate. Baseline Raman spectra of intact vertebrae and femurs from control mice were obtained prior to analyses of tumor burdened bones. Bones were stripped of the periosteum by lightly scraping with a scalpel and were wetted with Dulbecco's phosphate buffered saline. For the femurs, measurements from the distal metaphysis through the diaphysis to the proximal metaphysis were made at 2 mm intervals along the bone (Fig. 1A and B) resulting in 35 spectra per femur. For the spines, measurements were taken at approximately the center of lumbar (L1–L6), sacral (S1–S4), and caudal (C1–C2) vertebrae (Fig. 1A and B) resulting in 60 spectra per vertebral column. A home-built fiber-probe based Raman spectroscopy system was used to record the spectral profiles with 300s exposure time.^33^ Briefly, an 830 nm diode laser (Process Instruments, Salt Lake City, UT) was used as the excitation source. A lensed fiber-optic Raman bundled contact probe (EmVision LLC, FL) was used to deliver the excitation beam through its central fiber (200 μm core) and acquire the back-scattered light through an annular ring of optical fibers (300 μm core). The scattered light was transmitted by the fiber-probe to a f/1.8i spectrograph (Holospec, Kaiser Optical Systems, MI) while a thermoelectrically cooled deep-depletion CCD camera (PIXIS 400BR, Princeton Instruments, NJ) was used for spectral recording. The laser power delivered to the sample surface was maintained at approximately 15 mW. Measurements over the length of the bone were enabled by scanning the fiber-probe using motorized translation stages (T-LS13M, Zaber Technologies, Inc., Vancouver, Canada). Zaber console (open-source software) was employed to control the raster scan as well as maintain a constant distance above the bone surface. Five spectra were collected from each measurement site.

### Data analysis

The acquired spectra were imported into the MATLAB 2013a (Mathworks, Inc., Natick, MA) environment for further analysis. Spectra were processed to remove interference from cosmic rays. A background spectrum, obtained from measurements on the aluminum substrate, was subtracted from the acquired spectra to correct for extraneous optical fiber-probe background signal. The resultant spectra were divided by the white light response signal (obtained from a BaSO_4_ sample under tungsten halogen lamp illumination) to compensate for any spectral non-uniformity in the detection. Next, the fluorescence background was removed using an automated method outlined by the Berger laboratory.^34^ The resultant spectra were normalized to the intensity of the PO_4_^3–^ ν_1_ peak (*ca.* 958 cm^–1^).

In addition to peak height and full width at half maximum (FWHM) analyses, the Raman spectra were subjected to principal component analyses (PCA). Operating without any *a priori* knowledge of the samples, PCA seeks to project the spectral data onto a set of linearly uncorrelated (orthogonal) directions, *i.e.*, principal components (PC), such that the variance in the original data can be captured using only a few PCs.^35^ Furthermore, support vector machines (SVM) were used on the PC score inputs to develop a decision algorithm for spectroscopically predicting the progression of tumor-induced changes in the bone. SVM is a supervised learning model that is built on structural risk minimization concepts and can efficiently perform non-linear classification by implicitly mapping the inputs into high-dimensional feature spaces through a kernel. A radial basis function (RBF) with a Gaussian envelope was chosen as the kernel, and the kernel parameters were optimized based on an automated grid search algorithm.^36^–^38^ The output of the SVM-derived decision algorithm was validated against the known class labels, *i.e.*, the time point of the harvested bone sample. The performance of the algorithms was evaluated by determining the classification accuracy in a leave-one-spectrum-out cross-validation protocol.

## Results and discussion

**Fig. 2:**
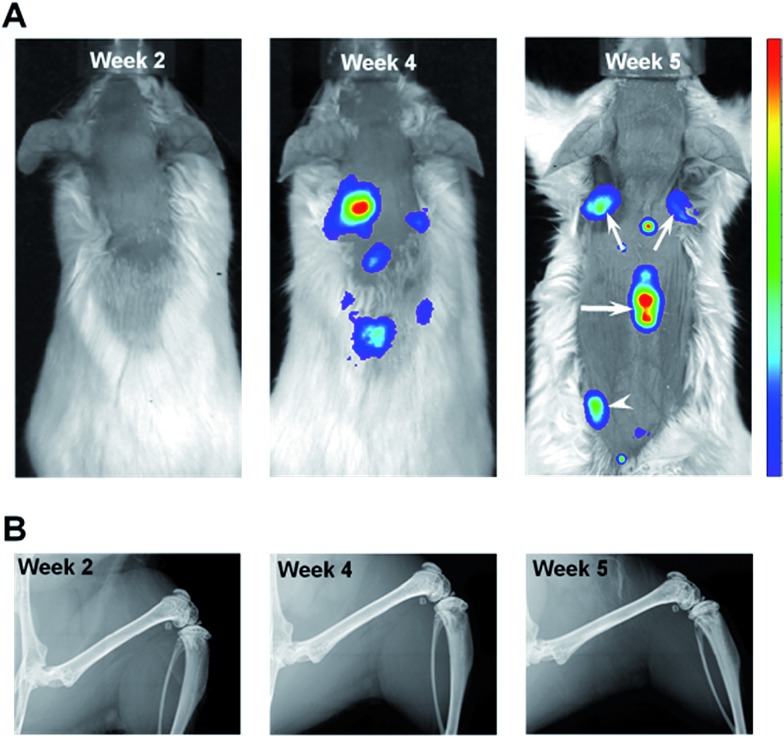
Live animal optical and X-ray imaging. (A) Fluorescence imaging of tdTomato signals from tumor-bearing mouse at week 2 (left panel) and tumor-bearing mice at 4 and 5 weeks post 435-tdT inoculations. In the week 5 image, fluorescent metastatic lesions in the scapulae (small arrows), lower thoracic-upper lumbar region of the spine (large arrow), and left proximal femur/pelvis region (arrowhead) were evident. Note, to better ascertain bone fluorescence signals, intense brain fluorescence was masked in the middle and right panel images. (B) X-ray images from a Faxitron X-ray scanner displaying the femur of the same mice shown in the corresponding panels in (A). No metastatic bone lesions were revealed in any of the X-ray images.

In order to track metastatic progression, we employed a variant of MDA-MB-435 breast cancer cells that have been engineered to constitutively express a bright red fluorescent protein.^32^ As shown in Fig. 2A, fluorescence signals from bone, *e.g.*, spine and scapulae, were recorded at weeks 4 and 5 and, at the latter time, fluorescence from the left pelvis/proximal femur region was also seen. No fluorescence signals were seen at week 2 (Fig. 2A). Thus, optical imaging could not detect (as shown below and by a previous group^28^) the nascent metastatic disease already present at week 2. In addition, as shown in Fig. 2B, no metastatic lesions were revealed by X-ray imaging of the femurs at any time point (or in the spine specimen, data not shown). This is consistent with the low sensitivity of X-ray imaging in the clinic where bone metastases are reported to be only detected in those cases where 30–75% of skeletal destruction has already occurred.^14^

This qualitative imaging data provided a means to establish the time course of metastatic progression and a confirmation that, similar to the clinical situation, X-ray imaging offers inadequate assessment of bone metastases, even when optical imaging indicated substantial tumor burden in bone (Fig. 2A, week 4 & 5). As such, we hypothesized that this model system would provide a suitable sample set to test whether Raman spectroscopy can detect early alterations of bone matrix integrity, which may be considered as a surrogate assessment of the underlying metastatic involvement.

We focused our Raman spectroscopic analyses on femurs and vertebrae as the former is the most affected long bone and spine represents one-third of the total skeletal metastases observed in breast cancer patients.^8^,^9^ Consequently, metastatic involvement at either or both sites accounts for a high proportion of SREs, and an early evaluation of metastases at these sites remains an unmet need. Assessing bone integrity using Raman spectroscopy provides specific quantitative evaluations of the bio-hydroxyapatite (mineral) component simultaneously with the associated collagen component (matrix).^21^,^23^,^25^,^26^ The mineral component is often calcium-deficient, and possesses carbonate substitutions with respect to stoichiometric hydroxyapatite. Recent investigations have extended the assessments of bone fragility and metastatic lesions using Raman spectroscopy to include in-depth evaluations of collagen integrity^16^,^18^,^24^ along with the contributions of proteoglycans, tissue water, and lipids^17^ as well as oxidative damage.^39^

**Fig. 3:**
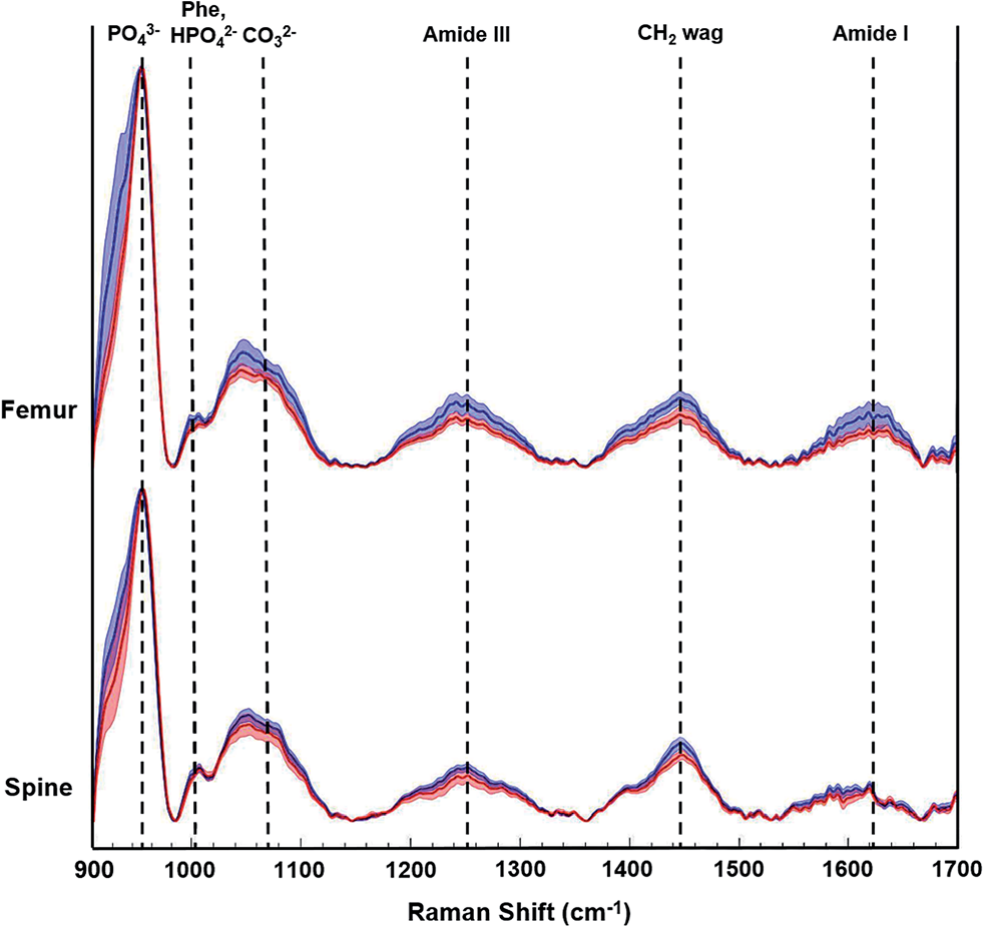
Representative Raman spectra acquired from metastatic breast cancer affected femurs and spines. Spectra (normalized to PO_4_^3–^ ν_1_ peak) were acquired from week 0 control group (blue tracings) and 5 weeks after tumor cell inoculations (red tracings). The solid lines depict the mean spectrum of each sample group with associated shadings representing the ±1 standard deviations (SD). Spectra are vertically offset for visualization purposes.

During this pilot study, we focused on peak signals associated with bio-hydroxyapatite to collagen matrix content, mineral crystallinity, and carbonation to derive specific quantitative distinctions between control and tumor-bearing bones. Fig. 3 shows representative Raman spectra from femurs and spines from a non-tumor bearing normal control mouse (blue tracings) and a tumor-bearing mouse (red tracings) sacrificed 5 weeks after tumor cell inoculations. The distinctive Raman peaks at *ca.* 958, 1004, 1070, 1250 and 1450 cm^–1^ correspond to the vibrational modes of phosphate ν_1_, phenylalanine (Phe) with potential contributions from monohydrogen phosphate, carbonate, amide-III and CH_2_ wag, respectively. We also observe the amide-I feature in the 1630–1656 cm^–1^ region. We calculated the mineral-to-matrix content as the phosphate ν_1_/amide I ratio, and phosphate-to-carbonate level (*i.e.* a marker of carbonate substitution) by the phosphate ν_1_/carbonate ratio. The degree of bone remodeling was estimated as the carbonate ν_1_/amide I ratio (since the carbonate-to-matrix ratio has been associated with increased risk of fracture), and mineral crystallinity as the reciprocal of full-width at half maximum (FWHM) of phosphate ν_1_ peak.^16^,^21^,^23^,^26^

**Fig. 4:**
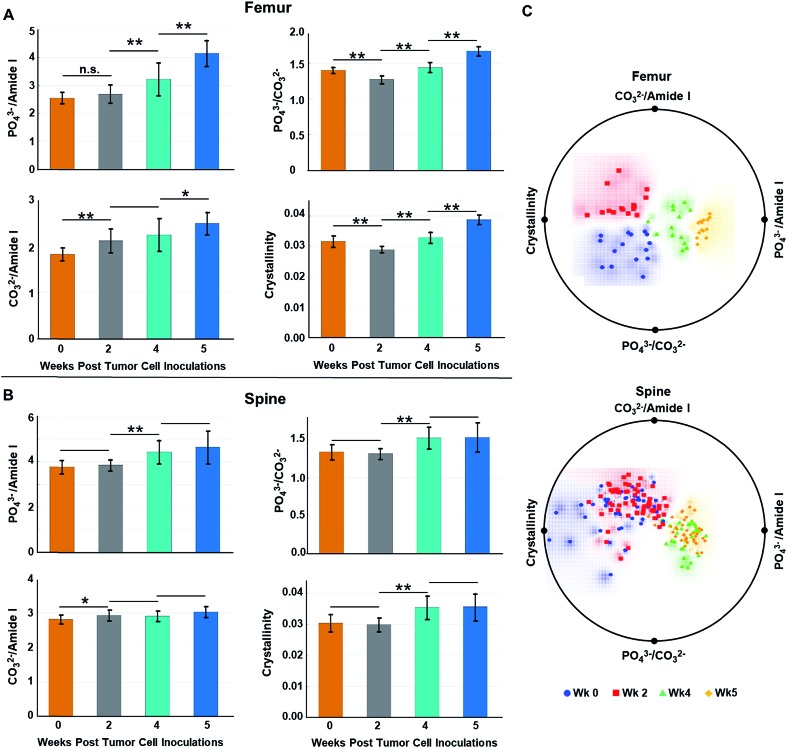
Raman spectra-derived metrics of bone compositional changes at each week of the study and corresponding radial visualization plots. Characteristics analyzed were: collagen mineralization as the PO_4_^3–^/amide I (phosphate ν_1_/amide I) ratio, phosphate-to-carbonate ratio: PO_4_^3–^/CO_3_^2–^ (phosphate ν_1_/carbonate) ratio, remodelling as the CO_3_^2–^/amide I ratio, and mineral crystallinity from 1/FWHM PO_4_^3–^ (1/FWHM phosphate ν_1_ peak) calculations. Relative to week 0, average compositional changes of (A) femurs and (B) spines at 2, 4, and 5 weeks post tumor cell inoculations were quantified. Error bars = ± 1 SD. (n,s, denotes not significant, *p < 0.05, **p < 0.005). (C) Distinct clustering of the spectral data corresponding to each week was revealed in the case of the femur analyses while two clusters emerged in the analysis of the spine data, namely, an early stage cluster: week 0 + week 2 and a late stage cluster: week 4 + week 5. Blue circles = week 0, red squares = week 2, green triangles = week 4, and orange diamond = week 5.

Fig. 4A and B show the (length-averaged) changes that occurred in these ratios in femurs and spines, respectively, during disease progression over the 5 week time course of the study. Relative to week 0, there was a progressive increase in the phosphate ν_1_/amide I ratio (Fig. 4A and B), as the tumor burden increased over time. This can be interpreted as being indicative of an increase in mineral density, which has previously been shown to be strongly negatively correlated to bone toughness.^23^ Alternatively, within the context of this osteolytic metastatic model, we need to also consider the possibility that both PO_4_^3–^ and type I collagen are decreasing with the degradation of collagen, especially by week 4 & 5, surpassing the loss of PO_4_^3–^ mineral content. Such a scenario reflects the fact that the colonizing tumor cells stimulate maturation of osteoclasts and hence bone resorption. This occurs by osteoclast-modulated acidification (∼pH 4.5) of the resorption area that dissolves mineral and simultaneously releases of cathepsin K thereby degrading the organic bone matrix.^40^ Stromal acidification would have also been enhanced by the proximal metabolic activities of the proliferating cancer cells, which generate low extracellular pH.^40^ It has been shown that metabolic acidification causes large releases of bio-hydroxyapatite mineral from bone,^41^ and such acidification may contribute to and increase the loss in PO_4_^3–^. At the same time, stromal cell and tumor cell production of matrix metalloproteinases could be expected to extend matrix degradation beyond resorption sites.^7^,^42^,^43^ This degradation of collagen has been recorded as blood borne type I collagen fragments, which are biomarkers of bone metastases.^44^ In addition, over the time course of the study, one expects an increasing loss of collagen regeneration by osteoblasts because expression of their matrix generating genes are known to be inhibited by acidification. Moreover, osteoblasts undergo apoptosis during cancer colonization,^28^,^29^,^41^ which would curtail bone formation. Similar to the consideration that an increase in mineral density weakens bone, loss of both mineral and matrix components during increasing losses of collagen would be expected to compromise the mechanical properties of the bone.^45^

Relative to week 0, changes in the carbonate substitution (PO_4_^3–^ ν_1_/CO_3_^2–^ ratio) do not exhibit a consistent trend. The ratio proceeds through an increase in CO_3_^2–^ substitution at week 2, which is more pronounced in the femurs relative to the spine (compare Fig. 4A to B), and then to decreases at weeks 4 & 5 (Fig. 4A and B). The increase at week 2 is consistent with previously reported results at a week 3 time point.^16^ This type of substitution has been shown to decrease crystallinity size, which generates defects in the apatite matrix and weakens the bone.^23^ The relatively high decrease in CO_3_^2–^ substitutions by week 5 of metastatic progression likely reflects high levels of osteolytic activity, *i.e.*, mineral dissolution, and concomitant utilization of released HCO_3_^–^ by the rapidly growing tumor mass as a means to neutralize intracellular pH.^46^

Throughout the course of metastatic progression in femurs as well as in spine, increased CO_3_^2–^/amide I ratios relative to week 0 were recorded (Fig. 4A and B). Similar to our discussion for the phosphate-to-matrix ratio, the progressive increase in the CO_3_^2–^/amide I ratios in femurs possibly reflects unchecked degradation/loss of collagen that exceeded the rate of loss of CO_3_^2–^. Such increases in CO_3_^2–^/amide I ratios has been shown to be associated with increased fracture risk in the literature.^47^

Relative to week 0, the bio-hydroxyapatite crystallinity (1/FWHM of PO_4_^3–^ ν_1_) decreased in femurs and was unchanged in spine at week 2 but thereafter increased in all bone samples (Fig. 4A and B). The decrease at week 2 is consistent with reported results at a week 3 time point^16^ and, as is generally the case, mirrors the increases in CO_3_^2–^ substitution (*i.e.*, decreases in PO_4_^3–^ ν_1_/CO_3_^2–^ ratio) at this time point.^48^ Replacement of phosphate with carbonate at week 2 increases the number of defects in the bio-hydroxyapatite lattice and reduces the crystallinity.^25^ Likewise, increases of crystallinity at the later time points parallels the decreases in carbonate content and may reflect an anticipated accelerated bone turnover during osteolytic metastatic progression.^48^ In addition, increases in crystallinity is likely an indication of retarded bone formation with a generation of larger thinner out-of-alignment crystallites rather than production of smaller younger crystals resulting in increased fragility.^17^,^48^ Increases of crystallinity have also been previously reported at a week 4 time point for MDA-MB-435 breast cancer cell metastases. While earlier time points were not evaluated, the authors suggested, given that a metastatic bone prostate model revealed decreases in crystallinity,^49^ that this spectral marker may differ with different primary tumors.^26^ However, our data indicate that, at early time points, decreased crystallinity can be associated with bone metastasis from breast cancer, suggesting that both the progression of metastasis and primary tumor type may impact changes in bone crystallinity.

We also performed radial visualization of the metrics generated from the femur and spine to assess the degree of inter- *vs.* intra-time point variability across the tested mice specimen. Fig. 4C shows distinct clustering patterns for the data points corresponding to the femurs from each week of evaluation. Evidently, crystallinity and the PO_4_^3–^/amide I ratios account for majority of the differential clustering of the femur data. For the spine measurements, we observed overlap between the week 0 and week 2 (early stage) as well as week 4 and week 5 (late stage) data points. Nevertheless, a clear distinction between the early and late stages of disease progression was revealed for both the femur and spine samples.

**Fig. 5:**
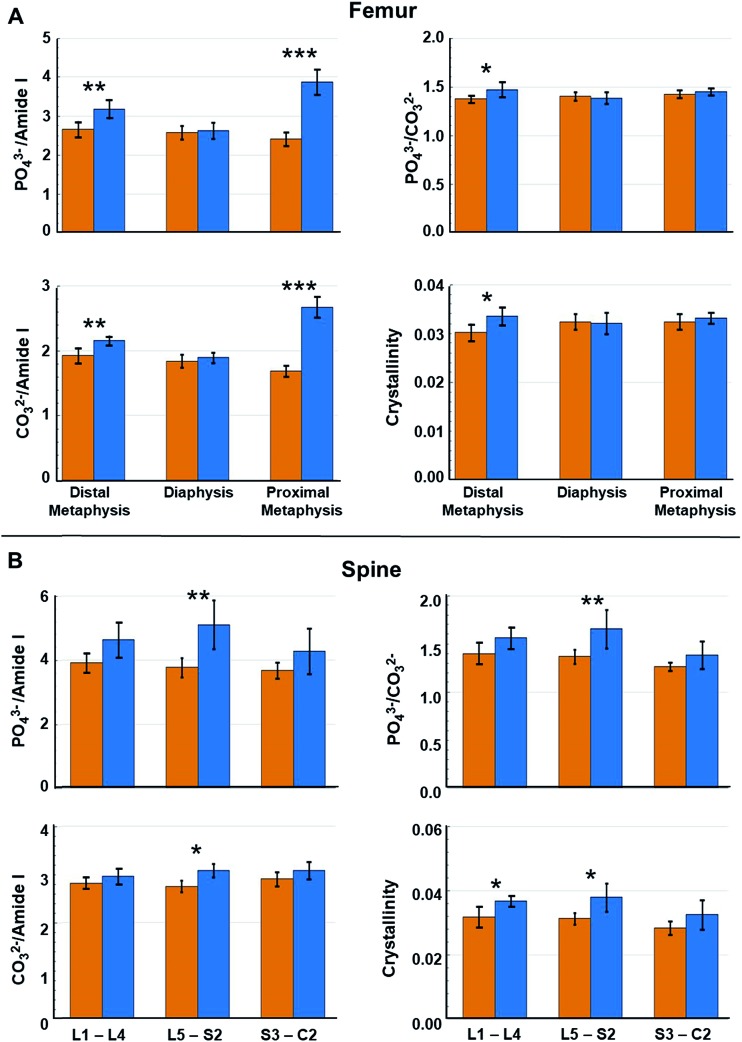
Raman spectral-derived metrics of bone compositional changes as a function of the position of the measurements on the bone. (A) Relative to week 0, average compositional changes (see Fig. 4) at the distal metaphysis, diaphysis, and proximal metaphysis of femurs. (B) Relative to week 0, compositional changes at lumbar vertebrae (L1–L4), lumbar–sacral vertebrae (L5–S2), and sacral–caudal vertebrae (S3–C2) of spines. Orange bar = week 0 and blue bar = week 4. Error bars = ± 1 SD. (*p < 0.05, **p < 0.01, ***p < 0.001).

However, as metastasis to long bones is known to vary along their lengths,^50^ we reasoned that alterations in specific regions of the femurs may have been masked within the averaged values. Fig. 5A indicates that changes in the computed metrics were more pronounced in the metaphysis of femurs and minimal changes in the spectral signatures/biochemical composition of diaphysis were registered. This is consistent with the known propensity of metastasizing tumor cells to colonize the highly vascularized remodeling niche of the metaphysis.^9^,^26^,^28^ In contrast to the femur, Fig. 5B indicates changes in bone compositional markers occur more uniformly throughout the length of vertebrae assessed. Yet, changes in bone composition were higher in vertebrae of the L5–S2 lumbar–sacral region, which is consistent with previous assessment of high tumor burden in these locations in this model system.^27^ Therefore, including measurements of vertebrae below the pelvic region may skew the average values. Notably, the portion of spine analyzed in this model system broadly parallels clinical disease as the lumbar-sacral region is the second most frequent site of spinal compression due to metastatic disease in patients.^51^

To further assess the utility of the spectral information in recognizing progressive stages of metastatic bone alterations, SVM-derived decision algorithm was developed on the principal component (PC) scores. Table 1 shows classification accuracies of metastatic involvement at different regions of the femurs across the five weeks of study. The overall classification accuracy obtained for the SVM-derived decision algorithm was found to be 93.6% with the classification accuracy for any combination of site and time point being ≥83%. Expectedly, the classification of diaphysis exhibited the lowest accuracy when compared to that of the metaphyseal regions.

**Table 1:** Classification results for the SVM-derived decision algorithm as a function of time point and location in the femur

Site	Week 0	Week 2	Week 4	Week 5	Average
Distal metaphysis	100%	95%	95%	85%	93.8%
Diaphysis	100%	87%	83%	93%	90.8%
Proximal metaphysis	100%	100%	95%	90%	96.3%

To ensure the robustness of this decision algorithm, we also conducted a negative control study where the spectra were randomly assigned to sites and time points, irrespective of their true identity.^52^ In this control study, a maximum average accuracy of *ca.* 26% was achieved over 20 iterations underscoring the reliability of the actual SVM-derived decision model in discerning subtle, but consistent, metastasis driven changes during the 5 week course the study.

Additionally, by restricting the analyses to only the prominent biomarkers noted above, we developed another decision algorithm to classify the femur sites at different time points (Table 2). High classification accuracies were obtained for the analyses using selected Raman features, despite utilization of only 27% of the entire spectral information. In particular, the distal and proximal metaphysis largely maintained the same accuracy levels as the full spectrum decision model (Table 1), while the misclassification rates were significantly higher for diaphysis. Also, as a maximum rate of osteolysis was approached (week 4–5), a slightly higher misclassification between week 4 and week 5 groups was obtained, especially for the proximal metaphysis. Corresponding classification results of spine are shown in ESI (Table S1†). One notable feature in the classification analyses for the spine is the inability of the SVM decision model to accurately distinguish between week 0 and week 2 cases (that shows up as a reduction in classification accuracy at week 0, particularly for the caudal vertebras) indicating that metastasis-induced compositional changes may not occur as early as in the femurs.

**Table 2:** Classification results for the SVM-derived decision algorithm using only the selected spectral features as a function of time point and location in the femur

Site	Week 0	Week 2	Week 4	Week 5	Average
Distal metaphysis	100%	100%	90%	90%	93.8%
Diaphysis	100%	83%	67%	76%	79.8%
Proximal metaphysis	100%	100%	90%	70%	90.0%

**Fig. 6:**
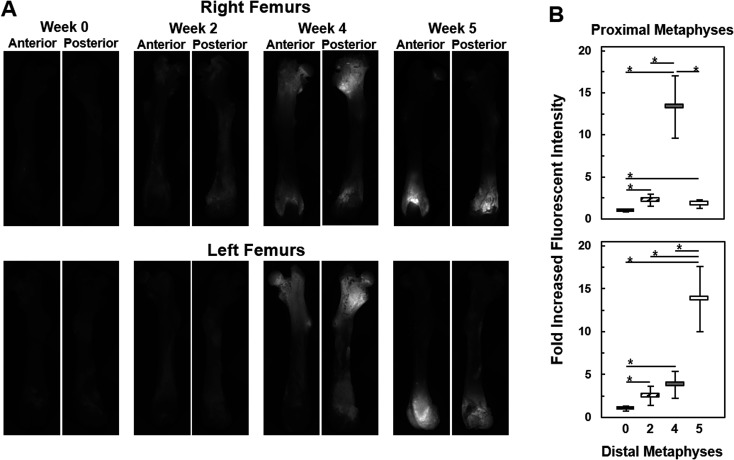
Fluorescent imaging based assessment of the metastatic lesions in femurs. (A) Fluorescence images of anterior and posterior views of right (top panels) and left (bottom panels) femurs from each week (0, 2, 4, 5) of the study. Autofluorescence was low (week 0 images), metastasis specific fluorescent signals from tdT-435 cells within the metaphysis regions was relatively weak at week 2 and much more intense at week 4 and 5. (B) Fold increases in fluorescent intensities from the metaphysis regions of femurs in (A) relative to week 0 autofluorescence as well as between weeks as determined by the semi-quantitative measurements. Error bars = ± 1 SD. Two-tailed Students *t*-test was employed for evaluating statistical significance (asterisk depicts *p* < 0.05).

Once the Raman spectroscopic analyses were completed on femurs, validation of the findings was sought from fluorescence imaging within different regions of the femurs. As seen in Fig. 6A, bright fluorescence from the tdTomato expressing tumor cells was readily recorded from metaphyseal regions of the femurs, especially at week 4 and 5, while diaphyseal regions showed no fluorescence signals. This is consistent with the results in Fig. 5A as well as the previously reported preferential location of metastasis to the metaphyseal regions of femurs.^28^Fig. 6B shows the assessment of the images in Fig. 6A in terms of fold increases in fluorescence intensities across metaphysis as a function of metastatic progression. We observe that as early as week 2 fluorescence intensities from within the metaphyseal regions was about 2.5-fold higher than the autofluorescent signals captured at week 0, *i.e.*, in femurs without tumor involvement. By week 4 the fluorescence intensity from the distal metaphysis was about *ca.* 3–4-fold higher than autofluorescence (week 0) while at the proximal metaphysis it was about 13-fold higher. A reverse trend was seen in the week 5 case where the fluorescence intensity was about 13-fold higher than background in the distal metaphysis while the proximal metaphysis harbored low amounts of tumor cells as reflected in a fluorescence intensity that was only about 1.5–2-fold higher than the autofluorescence of week 0. Except for comparisons between week 2 and week 4 distal metaphysis and week 2 and week 5 proximal metaphysis, fold increases in fluorescence intensities of each week of metastatic progression were all significant relative to week 0 as well as between weeks (two-tailed student *t*-test, asterisk: *p* < 0.05). Overall, fluorescence imaging observations were consistent with the quantitative Raman spectroscopic analyses, and provided an understanding of the differential degree of bone deterioration as a function of increasing metastatic tumor involvement over time. This imaging also reflects the heterogeneity and stochastic nature of metastatic progression, where, in this model system, metastasis to femur regions in different subjects can exhibit regional tumor burden differences; *e.g.*, week 4 or 5 femurs with dissimilar metastatic burdens at different ends of the femurs.

## Conclusions

In this pilot study, we demonstrated that Raman spectroscopy has the capability to detect biochemical changes to the structure of bones associated with cancer metastasis without *a priori* imaging or pathological knowledge of lesion location. Raman spectroscopy was able to identify sites of early tumor involvement, which points towards a potential diagnostic for early intervention not only to treat the advancing tumor but also to further assess these sites to circumvent pathological fractures. Hence, it may be possible to build an accurate risk assessment tool using Raman spectroscopy in conjunction with microCT or MRI. Along these lines, we showed that spectral changes emanating from the variations of specific spectral features can be utilized to construct decision algorithms with high diagnostic power.

Ultimately, we envision longitudinal studies will contribute to our understanding of molecular changes that indicate metastatic involvement at early stages. Movement towards bringing real time, fully noninvasive Raman spectroscopic assessments of bone to the clinic has been in progress in other laboratories, and impressive advances in instrumentation^53^ and data processing^15^ have been demonstrated. Together, such developments set the stage for future *in vivo* application of Raman spectroscopy for assessment of metastatic progression in bone and of fracture risk.

## Conflicts of interest

The authors disclose no potential conflicts of interest.

## Supplementary Material

Supplementary information

## References

[cit1] Siegel R. L., Miller K. D., Jemal A. (2017). Ca-Cancer J. Clin..

[cit2] DeSantis C. E., Lin C. C., Mariotto A. B., Siegel R. L., Stein K. D., Kramer J. L. (2014). Ca-Cancer J. Clin..

[cit3] Berman A. T., Thukral A. D., Hwang W. T., Solin L. J., Vapiwala N. (2013). Clin. Breast Cancer.

[cit4] Steinauer K., Huang D. J., Eppenberger-Castori S., Amann E., Guth U. (2014). J. Bone. Oncol..

[cit5] Oster G., Lamerato L., Glass A. G., Richert-Boe K. E., Lopez A., Chung K. (2013). Support Care Cancer.

[cit6] Fontanella C., Fanotto V., Rihawi K., Aprile G., Puglisi F. (2015). Clin. Exp. Metastasis.

[cit7] Liu B., Cui J., Sun J., Li J., Han X., Guo J. (2016). Mol. Med. Rep..

[cit8] Lin J., Goldstein L., Nesbit A., Chen M. Y. (2016). World Neurosurg..

[cit9] Märdian S., Schaser K. D., Ruppert M., Melcher I., Haas N. P., Schwabe P. (2015). Acta Chir. Orthop. Traumatol. Cech..

[cit10] Jambor I., Kuisma A., Ramadan S., Huovinen R., Sandell M., Kajander S. (2016). Acta Oncol..

[cit11] Kannivelu A., Loke K. S., Kok T. Y., Osmany S. Y., Ali S. Z., Suat-Jin L. (2014). Semin. Musculoskelet Radiol..

[cit12] Damron T. A., Nazarian A., Entezari V., Brown C., Grant W., Calderon N. (2016). Clin. Orthop. Relat. Res..

[cit13] Bolotin H. H. (2007). Bone.

[cit14] Snyder B. D., Cordio M. A., Nazarian A., Kwak S. D., Chang D. J., Entezari V. (2009). Clin. Cancer Res..

[cit15] Buckley K., Kerns J. G., Vinton J., Gikas P. D., Smith C., Parker A. W. (2015). J. Raman Spectrosc..

[cit16] Burke M. V., Atkins A., Akens M., Willett T. L., Whyne C. M. (2016). J. Orthop. Res..

[cit17] Paschalis E. P., Gamsjaeger S., Hassler N., Klaushofer K., Burr D. (2017). Bone.

[cit18] Unal M., Akkus O. (2015). Bone.

[cit19] Feng X., Moy A. J., Markey M. K., Fox M. C., Reichenberg J. S. and Tunnell J. W., Biophysical basis for noninvasive skin cancer detection using Raman spectroscopy, SPIE BiOS. International Society for Optics and Photonics, 2016, p. 97040C, 10.1117/12.2209421.

[cit20] Mahadevan-Jansen A., Richards-Kortum R. R. (1996). J. Biomed. Opt..

[cit21] Mandair G. S., Morris M. D. (2015). BoneKEy Rep..

[cit22] Bi X., Patil C. A., Lynch C. C., Pharr G. M., Mahadevan-Jansen A., Nyman J. S. (2011). J. Biomech.

[cit23] Inzana J. A., Maher J. R., Takahata M., Schwarz E. M., Berger A. J., Awad H. A. (2013). J. Biomech.

[cit24] McNerny E. M., Gong B., Morris M. D., Kohn D. H. (2015). J. Bone Miner. Res..

[cit25] Bi X., Sterling J. A., Merkel A. R., Perrien D. S., Nyman J. S., Mahadevan-Jansen A. (2013). Bone.

[cit26] Ding H., Nyman J. S., Sterling J. A., Perrien D. S., Mahadevan-Jansen A., Bi X. (2014). J. Biomed. Opt..

[cit27] Harms J. F., Welch D. R. (2003). Clin. Exp. Metastasis.

[cit28] Phadke P. A., Mercer R. R., Harms J. F., Jia Y., Frost A. R., Jewell J. L. (2006). Clin. Cancer Res..

[cit29] Mastro A. M., Gay C. V., Welch D. R., Donahue H. J., Jewell J., Mercer R. (2004). J. Cell. Biochem..

[cit30] Matousek P., Stone N. (2016). Chem. Soc. Rev..

[cit31] Brinkley B. R., Beall P. T., Wible L. J., Mace M. L., Turner D. S., Cailleau R. M. (1980). Cancer Res..

[cit32] Winnard Jr P. T., Kluth J. B., Raman V. (2006). Neoplasia.

[cit33] Paidi S. K., Siddhanta S., Strouse R., McGivney J. B., Larkin C., Barman I. (2016). Anal. Chem..

[cit34] Beier B. D., Berger A. J. (2009). Analyst.

[cit35] Ringner M. (2008). Nat. Biotechnol..

[cit36] Genton M. G. (2001). J. Mach. Learn. Res..

[cit37] Suykens J. A., Van Gestel T., Vandewalle J., De Moor B. (2003). IEEE Trans. Neural Network.

[cit38] Pelckmans K., Suykens J. A., Van Gestel T., De Brabanter J., Lukas L. and Hamers B., et al., LS-SVMlab: a matlab/c toolbox for least squares support vector machines, Tutorial. KULeuven-ESAT, Leuven, Belgium, 2002, vol. 142, pp. 1–2.

[cit39] Rubin M. R., Paschalis E. P., Poundarik A., Sroga G. E., McMahon D. J., Gamsjaeger S. (2016). PLoS One.

[cit40] Yoneda T., Hiasa M., Nagata Y., Okui T., White F. A. (2015). BoneKEy Rep..

[cit41] Bushinsky D. A., Chabala J. M., Gavrilov K. L., Levi-Setti R. (1999). Am. J. Physiol..

[cit42] Dutta A., Li J., Lu H., Akech J., Pratap J., Wang T. (2014). Cancer Res..

[cit43] Shimamura T., Amizuka N., Li M., Freitas P. H., White J. H., Henderson J. E. (2005). Biomed. Res..

[cit44] Dean-Colomb W., Hess K. R., Young E., Gornet T. G., Handy B. C., Moulder S. L. (2013). Breast Cancer Res. Treat..

[cit45] Depalle B., Qin Z., Shefelbine S. J., Buehler M. J. (2015). J. Mech. Behav. Biomed. Mater..

[cit46] Lee S., Mele M., Vahl P., Christiansen P. M., Jensen V. E., Boedtkjer E. (2015). Pfluegers Arch..

[cit47] Boskey A. L. (2013). BoneKEy Rep..

[cit48] Gong B., Oest M. E., Mann K. A., Damron T. A., Morris M. D. (2013). Bone.

[cit49] Esmonde-White K. A., Sottnik J., Morris M. and Keller E., Raman spectroscopy of bone metastasis, in Photonic Therapeutics and Diagnostics Viii, Pts 1 and 2, Proceedings of SPIE, ed. N. Kollias, B. Choi, H. Zeng, H. W. Kang, B. E. Knudsen and B. J. F. Wong, et al., Spie-Int Soc Optical Engineering, Bellingham, vol. 8207, 2012.

[cit50] Lynch M. E., Fischbach C. (2014). Adv. Drug Delivery Rev..

[cit51] Prasad D., Schiff D. (2005). Lancet Oncol..

[cit52] Winnard P. T., Zhang C., Vesuna F., Kang J. W., Garry J., Dasari R. R. (2017). Oncotarget.

[cit53] Demers J. L., Esmonde-White F. W., Esmonde-White K. A., Morris M. D., Pogue B. W. (2015). Biomed. Opt. Express.

