# Supplementary material for: Label-free Raman spectroscopy provides early determination and precise localization of breast cancer-colonized bone alterations
Source: Chem Sci. 2017 Nov 15;9(3):743–53. doi: 10.1039/c7sc02905e (PMC5869989; doi:10.1039/c7sc02905e)
Supplement: Supplementary file 1 [file SC-009-C7SC02905E-s001.pdf]

Electronic Supplementary Material (ESI) for Chemical Science.
This journal is © The Royal Society of Chemistry 2017

Supplementary Information

Label-free Raman spectroscopy provides early determination and precise localization of breast cancer-colonized bone alterations

Chi Zhang[1†], Paul T Winnard Jr.[2†], Sidarth Dasari[3], Scott L Kominsky[4], Michele Doucet[4], Swaathi Jayaraman[4], Venu Raman[2, 5,*], Ishan Barman[1, 5*]

[1]Department of Mechanical Engineering, Johns Hopkins University, Baltimore, MD, USA.
[2]Division of Cancer Imaging Research, Russell H. Morgan Department of Radiology and Radiological Science, Johns Hopkins University School of Medicine, Baltimore, MD, USA.
[3]Indiana University School of Medicine, Indianapolis, IN, USA.
[4]Department of Orthopaedic Surgery, Johns Hopkins University School of Medicine, Baltimore, MD, USA.
[5]Department of Oncology, Johns Hopkins University School of Medicine, Baltimore, MD, USA.

[†]These authors contributed equally to this work.

Running Title: Raman spectroscopic determination of early bone metastasis

Keywords: Raman spectroscopy, breast cancer, bone metastasis

***Correspondence:**
Ishan Barman
Johns Hopkins University
Whiting School of Engineering
Department of Mechanical Engineering
Latrobe Hall 103
Baltimore, MD 21218, USA.
Office Phone: 410-516-0656
E-mail: ibarman@jhu.edu

Venu Raman
The Johns Hopkins University School of Medicine
The Russell H. Morgan Department of Radiology and Radiological Science
Division of Cancer Imaging Research
720 Rutland Avenue, Rm 340 Traylor Building
Baltimore, MD, USA 21205
Office Phone: 410-955-7492
E-mail: vraman2@jhmi.edu

Conflict of Interest: The authors disclose no potential conflicts of interest.

Table S1A. Classification results for the SVM-derived decision algorithm as a function of time point and location in the spine

Site	Week 0	Week 2	Week 4	Week 5	Average
Lumbar vertebras	95%	100%	100%	100%	98.8%
Sacral vertebras	90%	100%	100%	90%	95.0%
Caudal vertebras	65%	100%	100%	100%	91.3%

Table S1B. Classification results for the SVM-derived decision algorithm using only the selected spectral features as a function of time point and location in the spine

Site	Week 0	Week 2	Week 4	Week 5	Average
Lumbar vertebras	85%	100%	95%	100%	95.0%
Sacral vertebras	90%	100%	100%	100%	97.5%
Caudal vertebras	65%	100%	100%	100%	91.3%
